# A new demand function graph: Analysis of retailer-to-individual customer product supply strategies under a non-essential demand pattern

**DOI:** 10.1371/journal.pone.0298381

**Published:** 2024-02-29

**Authors:** Zhiyi Zhuo, Shuhong Chen, Hong Yan, Yue He

**Affiliations:** 1 School of Business, Wuyi University, Wuyishan, Fujian, China; 2 School of Mathematics and Computer, Wuyi University, Wuyishan, Fujian, China; 3 School of Management, Zhejiang Shuren University, Hangzhou, Zhejiang, China; 4 Business School of Sichuan University, Chengdu, China; University of Naples Federico II: Universita degli Studi di Napoli Federico II, ITALY

## Abstract

The core objective of a successful product supply strategy is to determine the mechanism through which consumers’ psychological effects influence customer demand. As stated in the theory of supply and demand, a higher level of dynamic equilibrium should be formed in which demand drives supply and supply creates demand. There is a lack of systematic research in the literature on the identification of consumer goods demand attributes and the formation of influencing factors in consumer goods supply chains. In this paper, we use the literature on demand functions and product pricing functions to establish three mathematical models to study the factors that influence retailers in designing and planning product supply strategies for different customers under nonessential demand patterns and to solve the profit maximization problem. The results of numerical examples validate the validity of the model. The research results can help retailers develop different supply strategies according to different types of customers and different demand patterns, thereby improving business performance. The theoretical contribution of this study is the construction of value ranges and a demand function diagram for identifying consumer product demand attributes.

## 1 Introduction

In the field of consumer goods supply chains, customer demand plays a critical role in product price strategies, and eventually, a number of classic theoretical models on customer demand have been developed with fruitful results [1–3]. However, these classic demand models consider only a single decision variable and do not truly express how people make choices when faced with a dizzying array of goods. With the rapid development of the economy, the per capita disposable income of residents is also increasing when people are buying goods. In addition, a price factor of [4–6] often involves the influence of consumer psychology factors, such as customer experience, fashion and emotional factors [7–9].

Especially in recent years, with the popularization of the internet+ and artificial intelligence, new market formats in the internet+ era, such as online direct selling and live broadcast e-commerce, have become emerging business models and have shown strong vitality in the Chinese market, creating a new development pattern [10, 11]. Scholars generally believe that convenience and hedonic motivation are important determinants of the unusually strong vitality of emerging shopping behaviors such as live broadcast e-commerce [7, 8]. The main reason for this is that the physical characteristics conveyed by the experiment of substitute products and the values shared through instant interaction, as the two most important characteristics of live broadcast e-commerce, can effectively help consumers reduce uncertainty about products, thereby cultivating the trust of consumers with similar physical characteristics and values [12].

The basic characteristic of demand is the driving force behind the existence of products in the commodity economy. Whether it is the traditional real economy or the new economic model based on "Internet +", the ultimate goal of all business operators is to maximize profits [13, 14]. We believe that in a traditional economy, product price is the decisive factor affecting consumer demand and purchase patterns [4–6]. With the rapid rise of innovative business models such as mobile internet and artificial intelligence, people’s purchasing behaviour is influenced by consumer psychological factors such as the online marketing of live broadcasters [7–9]. This is because the mobile internet and big data have demonstrated a strong ability to meet high-level and new human needs; at the same time, they can also provide strong support in the realization of human social interaction and emotional needs, the construction of virtual worlds, and the connection between virtual and reality [15–17]. In this vein, how to meet the needs of consumers has become the core and key issue that enterprises must solve. We believe that the way to solve this problem is to design and plan a product supply strategy by exploring the attributes of consumer demand for products.

In the literature on this topic, Bendoly et al. [18] contend that supply chain inventory management decisions are based on the existence of uncertainty in both downstream (demand) and upstream (supply) aspects. The study revealed that the impact of supply and demand uncertainty on decision bias is more nuanced than the effect of each uncertainty alone on decision bias. Yu & Yan [19] discuss three selling strategies of sellers who produce and sell seasonal products to consumers under supply-demand uncertainty, full presale, partial presale, and nonpresale and develop a robust newsvendor model. The effects of supply and demand uncertainty and supply and demand correlations on these strategies are related to price discounts. Li [20] investigated the effect of reduced demand uncertainty in the case of two news retailers competing for product supply; they parameterized the level of uncertainty by means of mean retention spreads and studied the effect of reduced demand uncertainty on the equilibrium inventories of the two retailers and their expected profits. Yan [21] examined the impact of order quantities in supply chains when demand and/or supply uncertainty is present. The findings suggest that in a hybrid model comprising two types of uncertainty, supply uncertainty has a greater influence on the implementation of order quantities than does demand uncertainty. Srivastava et al. [22] determine the inventory replenishment policy when the demand rate is a function of the inventory space allocated to the products on the retail shelves. It was found that when the demand rate is a function of the initial allocation and the level of displayed inventory, an inventory policy that maximizes expected profit can be determined. Pei et al. [23] proposed a new uncertainty distribution set to describe the uncertainty of demand distribution based on fuzzy theory and developed a new two-channel green supply chain model with a robust pricing game. The study demonstrates the effect of demand ambiguity on manufacturers’ equilibrium channel selection strategies through numerical analyses and comparisons.

However, it is worth noting that while demand-related issues have been extensively explored in the academic study of marketing and supply chain strategy, most study results are arrived at under the circumstance of demand uncertainty and do not consider The demand attributes behind the products. The purpose of this paper is to study the influence of consumer psychological effects on customer demand. The theoretical basis of this paper comes from Marcuse’s real and false needs theory [24, 25].

Through the ideology of a developed capitalist society, Marcuse found that the materiality of products has led to contemporary product supply, in which consumer psychology is the main factor affecting the purchasing power of consumers [24, 25]. Marcuse further believes that most current consumer needs (such as rest, entertainment, and advertising) are caused by consumer psychology and can be classified as spurious demands. False demand is a purely mental or psychological phenomenon that is the product of consumer psychology and social culture. Marcuse believed that ideology was the leading factor that increasingly affected the purchasing power of consumers and that the demand that was influenced by the external environment was false [26, 27].

In regard to the supply chain of consumer goods, Marcuse’s study has several shortcomings. For example, luxury goods (such as jewellery) are a type of product that modern city dwellers like to buy. They are a spiritual product that is purely aesthetic. This type of product has nothing to do with people’s survival needs. According to Marcuse’s study, this is undoubtedly a false demand [24, 25]. However, from the point of view of consumer psychology, although it is true that people’s purchase of luxury goods is a kind of false demand, the transactional behaviour of buying luxury goods is real, and from the point of view of the transaction, it is certainly real demand. In a sense, false demand is essentially real demand. Therefore, we need to redefine customer demand in the consumer goods supply chain.

We believe that there are two basic models of customer demand in the consumer goods supply chain: essential demand patterns and nonessential demand patterns. Essential demand patterns indicate that consumer needs are based on what consumers truly need, i.e., the part of the product marketing process that belongs to the intrinsic functions of products, while nonessential demand patterns are the part of the product marketing process that is sublimated to the ideological field, i.e., the product marketing process. This part is influenced by external environmental factors such as economic benefits and advertising [28–32]. At present, with the continuous progress of social productivity and the continuous improvement of people’s living standards, the demand characteristics of consumers’ essential needs have been satisfied. The products in the market are more characteristic of nonessential demand patterns. Therefore, there is an urgent need to explore the influences of various unconscious or subconscious consumption habits on people’s essential development needs and motivations in the market supply and demand relationship, as well as the comprehensive influence mechanism of consumer psychology on customer needs. In this paper, we studied the design and planning of retailers’ supply strategies for individual customers under nonessential demand patterns. Focusing on the mechanisms by which consumer psychology influences customer demand, this study fills an important theoretical gap in the field of consumer goods supply chains and is highly innovative.

## 2 Variable functions

Customer demand is complex, and different people, backgrounds, economic strengths, social experiences, and cultures produce different customer needs. Research over the last few decades has shown that demand function modelling can be of great help in understanding customer demand in a commodity economy [33–37]. The demand function can be used as a way to regulate the existence and uniqueness of the optimal solution for profit maximization of various products, as well as a sufficient condition that can effectively show some properties of the optimal solution to find the optimal ordering strategy for the problem under consideration. On this basis, economic reasoning is carried out using linear demand and supply diagrams, and ultimately, mathematical models of economic reasoning are applied to investigate the interaction between mathematical logic and economics [38–40]. However, while these models and results have examined various aspects of the application of customer demand in a commodity economy, they have not considered the demand characteristics of consumer products.

### 2.1 Demand function

The objective of retailer product supply strategy (PST) problems based on customer needs is to find retailers to design and plan optimal supply plans for different types of customers, including individual and group customers, to maximize product profitability ^1^. When designing and creating a plan, the retailer must first consider the market equilibrium problem. If the quantity of products sold exceeds demand, there will be surplus products, and the remaining products will occupy inventory space. Such oversupply prevents firms from maximizing profits. Conversely, if the quantity sold is less than the consumer demand, the supply shortage will inevitably lead to the forgone profits not satisfying the demand from the remaining consumers who still have demand, which also prevents firms from maximizing profits.

We study such a simple PST, assuming that only one distribution model is considered, in which the manufacturer produces the product and makes wholesale sales to the retailer, which then sells the product to the individual final customer. We first design the variables included in the study and then construct a mathematical model of a retailer-personal customer PST under a non-essential demand pattern.

Petruzzi and Dada [41] studied the general demand function and assumed that the randomness of demand is independent of price. Therefore, the associated model could be constructed through addition or multiplication. Mills [42] defines the demand function as a model such as *D*(*p*,*ε*) = *y*(*p*)+ε. Karlin and Carr [43] used a multiplicative demand function of the form *D*(*p*,*ε*) = *y*(*p*)ε. In this case, *y*(*p*) is a function of the relation between demand and price and a random variable in the range [*A*,*B*]. Both *y*(*p*) models are common in the economics literature on price. The additive mode produces a static demand curve, and the multiplicative form produces an elastic demand curve. This model indicates that the shape of the market demand curve is also deterministic, while the market size is random. To ensure that demand is positive within a specific price range, let *A*>-*a* in the additive model and *A*>0 in the multiplicative model. However, in practice, *a* has a large variance in ε, and it is often necessary to employ an unbounded probability distribution such as a normal distribution to better approximate reality. In general, let *F*(ε) be the distribution function of ε; then,*F*(-∞) = 0,*F*(+∞) = 1, and *f*(ε) is the density function of ε. Moreover, we denote by *μ* and σ the expected and standard deviation of ε, respectively. For convenience, we consider only the additive model of demand.

Consider the demand function *D*(*p*,ε) = *y*(*p*)+ε, where

$$y(p)=a-bp,(a>0,b>0),\varepsilon ∼N(\mu ,\sigma^{2})$$ (1)

Then, according to Chen and Wang ^44^, it is assumed that the performance *U* of the product includes the utility function *u*(*x*) and the grade function *v*(*x*), thereby obtaining the product performance function:

$$U=w_{1}u+w_{2}v$$ (2)

$$w_{1}+w_{2}=1$$ (3)

where *w*_1_ represents the weight of the consumer’s utility function and *w*_2_ represents the weight of the consumer’s taste function. Then,

$$0\leq w_{1},w_{2}\leq 1$$ (4)

If *w*_1_ = 1, the product is purely practical, so the consumer’s demand for the product is real demand. If *w*_1_ = 0, the product has zero utility, which means that the consumer’s demand is nonessential. These are two extreme situations. Below, we analyse *w*_1_ to determine the weight ranges of the essential demand, nonessential demand and semiessential demand.

According to the maximization of the consumer’s expected utility, the use value is equal to 1, and the corresponding maximum demand intensity or reserve price is *p*_*m*_. Assume that the reserve price of the actual product increases linearly with the utility of the product. For products with utility value U ≤ 1, the consumer values the product at *p*_*m*_*U*, and the condition for the consumer to purchase is that the product price *P*satisfies *p*_*m*_*U*—*p* ≥ 0.

Then, by substituting Eqs (1) and (2) into Eq (3), the following can be solved:

$$1\geq U=w_{1}(u\text{-}v)+v\geq \frac{p}{p_{m}}$$ (5)

which can be rearranged as

$$w_{1}\geq \frac{\frac{p}{p_{m}}-v}{u-v},$$ (6)

and set (*u* > *v*) to make its critical point

$$w_{01}=\frac{\frac{p}{p_{m}}-v}{u-v}$$ (7)

**Fig 1:**
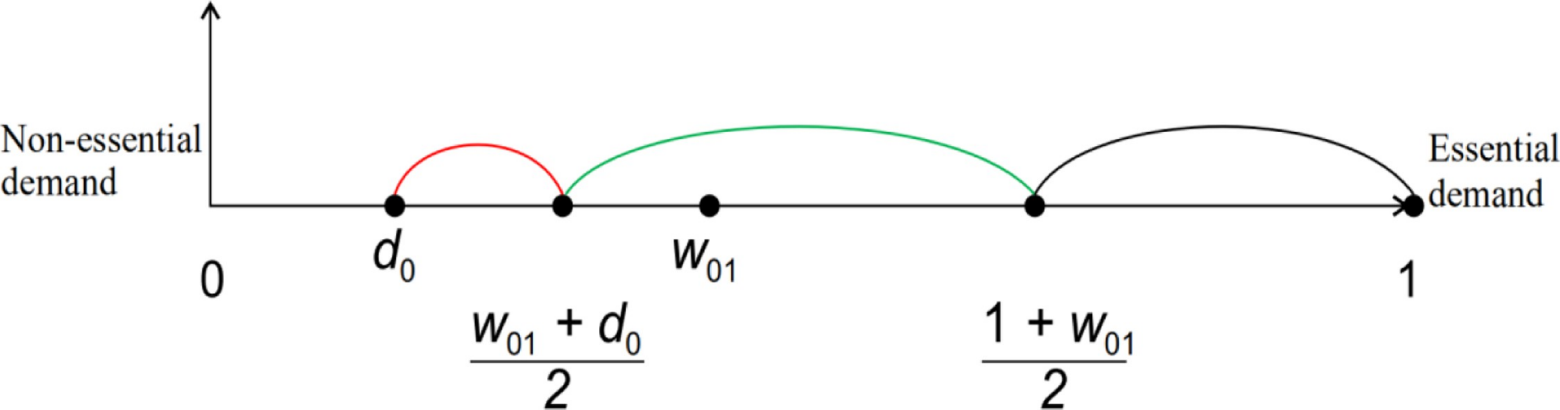
Demand function graph.

The minimum value of *w*_1_ is *d*_0_ < *w*_01_, as shown in Fig 1.

Thus, we propose the following definitions.

When $w_{1}\in \left[d_{0},\frac{w_{01}+d_{0}}{2}\right),$we have nonessential demand.

When $w_{1}\in \left[\frac{w_{01}+d_{0}}{2},\frac{1+w_{01}}{2}\right),$ we have semiessential demand.

When$w_{1}\in \left[\frac{1+w_{01}}{2},1\right]$we again have essential demand;

Unlike existing demand functions such as classical price-rigid and price-elastic demand, we do not distinguish between the three different demand patterns on the basis of the price of the product but rather on the basis of the context, motivation and psychological factors of the consumer at the time of purchase; i.e., more consideration is given to what is the main psychological purpose of the consumer in purchasing the product. In the consumer goods supply chain, the ideological component of customer demand is an important determinant of consumer purchasing power. Therefore, following the research objectives of this paper, we choose nonessential demand as our primary variable of interest.

### 2.2 Retailer’s profit function

In the operation of PSTs, the manufacturer must first wholesale the product to the retailer. If the quantity *q* of the wholesale product exceeds consumer demand, there will be surplus products, and the remaining products occupy inventory space. Therefore, it is impossible to achieve profit maximization in the presence of such oversupply. In contrast, if the quantity purchased is less than consumer demand, this supply shortage entails forgoing revenues from the remaining customers; thus, it is also impossible to maximize profits in this case.

When supply exceeds demand, set the purchased quantity at *q* and the quantity demanded at *pD*(*p*,ε), while the remaining quantity *q*-*D*(*p*,ε) is processed according to the cost per unit *h*. Here, *h* ≥ -*c* can take a negative value, which represents the residual of the unit product. Thus, in this case, the retailer’s profit is

$$\prod (p,p)=pD(p,\varepsilon )-cq-h\left.\left[q-D(p,\varepsilon )\right.\right]$$ (8)

Similarly, when supply is less than demand, if the purchased quantity is *q*, the selling price is *pq*, and the loss per unit is *S*. Here, the retailer’s profit is

$$\prod (q,p)=pq-cq-S\left.\left[D(p,\varepsilon )-q\right.\right]$$ (9)

Thus, we define the retailer’s profit as follows:

$$\prod (p,p)=\left\{\begin{array}{l} pD(p,\varepsilon )-cq-h\left[\left.q-D(p,\varepsilon )\right],q\geq D(p,\varepsilon )\right.\\ pq-cq-S\left[D\left(p,\varepsilon \right)-q\right],\;q<D\left(p,\varepsilon \right) \end{array}\right.$$ (10)

## 3 Parameter design

Using the above-described function, we construct a mathematical model of retailer-to-individual customer PST under the nonessential demand pattern. However, we first designed the parameters needed for modelling (Table 1):

**Table 1:** List of notations.

SYMBOL	MEANING
*α*	The discounted price per product in the off-invoice model
*p*	The retail price of the product set by the retailer
*q*	The quantity ordered by the retailer from the manufacturer
*u*	The practical performance of the product
*v*	The utility function of the product
*w*_1_	The weight on the practical performance of the product
*w*_2_	The weight on the utility function of the product
*d*_0_	The minimum weight on the practical performance of the product
*p*_*m*_	The product’s maximum performance or reserve price
*D*_*f*_	The non-essential demand function
Π_*R*_	The retailer’s profit
*I*	Individual customer

## 4 Mathematical model construction and analysis

In the consumer goods supply chain, it is common for manufacturers to offer trade promotions to retailers in the manufacturer-retailer supply chain, and there are various types of trade promotions, such as off-invoice, scan-back, unsold-discount and return credit [44–48]. In this section, we construct three mathematical models to represent the off-invoice mode, scan-back mode, and return credit mode for retailer-to-individual customer PSTs under the nonessential demand pattern.

Before modelling, we set the ranges of values for the relevant decision variables to [28–32]. By substituting the utility value into the demand function, the expression for the nonessential demand function becomes

Before modelling, we set the ranges of values for the relevant decision variables to ^28–32^. By substituting the utility value into the demand function, the expression for the non-essential demand function becomes

$$D_{F}=\frac{3}{2}(\frac{p-vp_{m}}{p_{m}(u-v)}-d_{0})(a-bp+\varepsilon ),a,b>0,\varepsilon \in U(\mu ,\sigma^{2})$$ (11)

In this case, ε obeys a normal distribution. To facilitate the study and maintain generalizability, we set ε to obey a uniformly distributed function in the interval [-(*a*-*bp*),*a*-*bp*].

Then,

$$f(\varepsilon )=\frac{1}{2(a-bp)},\varepsilon \in \left[\left.-(a-bp),a-bp\right]\right.$$ (12)

Below, we analyse the optimal purchase volume and optimal pricing scheme of retailers under the nonessential demand pattern and several supply modes.

### 4.1 Off-invoice mode

In this supply mode, the traditional promotion strategy is that the manufacturer supplies the retailer at a per-product discount of *α*, which is directly deducted from the purchase amount. Therefore, the actual wholesale price of the product for the retailer is *w*_0_ - *α*. In this case, if the retailer’s purchase amount is q ≤ *D*_*F*_, the supply is less than the demand, the obtained goods can be sold, and the retailer’s income is *pq*. If the retailer’s purchase amount is *q* > *D*_*F*_, demand is greater than supply, and the retailer’s income is pD_*F*_. We can obtain the retailer’s profit as follows:

$$\Pi_{R}=\left\{\begin{array}{l} -q(w_{0}-\alpha )+pq,\;q\leq D_{F}\\ -q(w_{0}-\alpha )+pD_{F},\;q>D_{F} \end{array}\right.$$ (13)

Using the expectation formula, the average profit earned by the retailer is:

$$\begin{array}{c} \Pi_{RF}=\int_{-\infty }^{q}\left(-q\left(w_{0}-\alpha \right)+pD_{F}\right)f(\varepsilon )d\varepsilon +\int_{q}^{+\infty }\left(-q\left(w_{0}-\alpha \right)+pq\right)f(\varepsilon )d\varepsilon \\ \begin{array}{l} =-q\left(w_{0}-\alpha \right)+\int_{0}^{q}p\frac{3}{2}\left[\frac{p-vp_{m}}{p_{m}(u-a)}-d_{0}\right]\varepsilon f(\varepsilon )d\varepsilon +\int_{q}^{2(a-bp)}qpf(\varepsilon )d\varepsilon \\ \begin{array}{c} =-q\left(w_{0}-\alpha \right)+\frac{3}{2}p\cdot \left(\frac{p-vp_{m}}{p_{m}(u-v)}-d_{0}\right)\int_{0}^{q}\varepsilon \cdot \frac{1}{2(a-bp)}d\varepsilon +pq\int_{q}^{2(a-bp)}\frac{1}{2(a-bp)}d\varepsilon \\ =\left(-w_{0}+\alpha +p\right)q+\frac{3pq^{2}}{8(a-bp)}\left[\frac{p-vp_{m}}{p_{m}(u-v)}-d_{0}\right]-\frac{pq^{2}}{2(a-bp)} \end{array} \end{array} \end{array}$$ (14)

In this supply mode, to obtain the retailer’s optimal purchase volume and optimal price we simply need to use the optimization method to derive the correlation function from the above formula and set its result equal to 0.

At this point, to obtain the retailer’s optimal pricing formula *p*,

$$\begin{array}{l} \frac{\partial \Pi_{RF}}{\partial p}=q+\frac{3q^{2}}{8\left(a-bp\right)}\left(\frac{2p-vp_{m}}{p_{m}\left(u-v\right)}-d_{0}\right)+\frac{3bq^{2}p}{8{\left(a-bp\right)}^{2}}\left(\frac{p-vp_{m}}{p_{m}\left(u-v\right)}-d_{0}\right)\\ -\frac{q^{2}}{2\left(a-bp\right)}-\frac{bpq^{2}}{2{\left(a-bp\right)}^{2}}=0 \end{array}$$ (15)

The solution for *p* is the retailer’s optimal price.

Then,

$$\frac{\partial \Pi_{RF}}{\partial q}=-w_{0}+\alpha +p+\frac{3pq}{4}\left(\frac{p-vp_{m}}{p_{m}(u-v)}-d_{0}\right)-\frac{pq}{\left(a-bp\right)}=0$$ (16)

The solution for *q* is the retailer’s optimal purchase amount.

### 4.2 Scan-back mode

In this supply mode, the manufacturer transfers some of her profits to the retailer; however, this share is not directly deducted at the time of purchase but rather after the retailer sells the product. Suppose that the manufacturer returns profit share *β* to the retailer based on the given wholesale price of *w* for each unit sold. Set the purchase quantity of the retailer at *q*; if the quantity purchased is greater than the quantity demanded, then the retailer’s income is (*p*+*β*)D_*F*_, where *p* is the retail price charged by the retailer. If the purchase quantity is *q*, the supply is less than the demand, and the retailer’s income is (p + *β*)*q*. We can obtain the retailer’s profit as follows:

$$\Pi_{RF}=\left\{\begin{array}{l} \text{-}w_{0}q+(p+\beta )q,q\leq D_{F}\\ \text{-}w_{0}q+(p+\beta )D_{F},q>D_{F} \end{array}\right.$$ (17)

Using the expectation formula, the average profit earned by the retailer is:

$$\begin{array}{l} \Pi_{RF}=\int_{q}^{+\infty }\left[\left.-w_{0}q+(p+\beta )q\right]\right.f(\varepsilon )d\varepsilon +\int_{\text{-}\infty }^{q}\left[\left.-w_{0}q+(p+\beta )D_{F}\right]\right.f(\varepsilon )d\varepsilon \\ =-w_{0}q+(p+\beta )q\int_{q}^{2\left(a-bp\right)}f(\varepsilon )d\varepsilon +(p+\beta )\int_{0}^{q}D_{F}f(\varepsilon )d\varepsilon \\ =-w_{0}q+(p+\beta )\int_{0}^{q}\frac{3}{2}\left[\left.\frac{p-vp_{m}}{p_{m}(u-v)}-d_{0}\right]\right.\varepsilon \cdot \frac{1}{2\left(a-bp\right)}d\varepsilon +(p+\beta )q\int_{q}^{2\left(a-bp\right)}\frac{1}{2\left(a-bp\right)}d\varepsilon \\ =-w_{0}q+(p+\beta )\cdot \frac{3}{2}\left[\left.\frac{p-vp_{m}}{p_{m}(u-v)}-d_{0}\right]\right.\cdot \frac{q^{2}}{4\left(a-bp\right)}+(p+\beta )q-\frac{\left(p+\beta \right)q^{2}}{2\left(a-bp\right)}\\ =\left(\text{-}w_{0}+p+\beta \right)q+\frac{3\left(p+\beta \right)q^{2}}{8\left(a-bp\right)}\left(\frac{p-vp_{m}}{p_{m}\left(u-v\right)}-d_{0}\right)-\frac{\left(p+\beta \right)q^{2}}{2\left(a-bp\right)} \end{array}$$ (18)

In this supply mode, to obtain the retailer’s optimal purchase volume and optimal price, we simply need to use the optimization method to derive the correlation function from the above formula and set its result equal to 0.

At this point, to obtain the retailer’s optimal pricing formula *p*,

$$\begin{array}{l} \frac{\partial \Pi_{RF}}{\partial p}=q+\frac{3q^{2}}{8\left(a-bp\right)}\left(\frac{2p-vp_{m}+\beta }{p_{m}\left(u-v\right)}-d_{0}\right)+\frac{3b\left(p+\beta \right)q^{2}}{8{\left(a-bp\right)}^{2}}\left(\frac{p-vp_{m}}{p_{m}\left(u-v\right)}-d_{0}\right)\\ -\frac{q^{2}}{2\left(a-bp\right)}-\frac{\left(p+\beta \right)bq^{2}}{2{\left(a-bp\right)}^{2}}=0 \end{array}$$ (19)

The solution for *p* is the retailer’s optimal price.

Then,

$$\frac{\partial \Pi_{RF}}{\partial q}=-w_{0}+p+\beta +\frac{3\left(p+\beta \right)q}{4\left(a-bp\right)}\left(\frac{p-vp_{m}}{p_{m}\left(u-v\right)}-d_{0}\right)-\frac{\left(p+\beta \right)q}{a-bp}=0$$ (20)

The solution for *q* is the retailer’s optimal purchase amount.

### 4.3 Return credit mode

In this supply mode, the manufacturer compensates the retailer for products that the latter failed to sell at discounted prices. In this case, the retailer purchases at unit price *w*_0_, and the purchased quantity is *q*. If the quantity demanded *D*_*F*_ is greater than the quantity purchased *q*, all of the products can be sold, assuming that the retail price is *p* and that the retailer’s sales amount is *pq*. If demand *D*_*F*_ is less than the quantity purchased *q*, then after demand is met, the remaining products cannot be sold. The manufacturer sets a recovery price of γ per unit of this unsold inventory, and the total recovery amount is *pD*_*F*_ + γ(*q*-*D*_*F*_). We obtain the retailer’s profit as follows:

$$\Pi_{RF}=\left\{\begin{array}{l} \text{-}w_{0}q+pq,q\leq D_{F}\\ \text{-}w_{0}q+pD_{F}+\gamma (q-D_{F}),q>D_{F} \end{array}\right.$$ (21)

Using the expectation formula, the average profit earned by the retailer is:

$$\begin{array}{l} \Pi_{RF}=\text{-}w_{0}q+\int_{0}^{q}\gamma (q-D_{F})f(\varepsilon )d\varepsilon +\int_{0}^{q}pD_{F}f(\varepsilon )d\varepsilon +\int_{q}^{2\left(a-bp\right)}pqf(\varepsilon )d\varepsilon \\ =\text{-}w_{0}q+\left(p-\gamma \right)\int_{0}^{q}D_{F}\left(\varepsilon \right)f\left(\varepsilon \right)d\varepsilon +\gamma q\int_{0}^{q}f\left(\varepsilon \right)d\varepsilon +pq\int_{q}^{2\left(a-bp\right)}f\left(\varepsilon \right)d\varepsilon \\ =\text{-}w_{0}q+\left(p-\gamma \right)\int_{0}^{q}\frac{3}{2}\left(\frac{p-vp_{m}}{p_{m}\left(u-v\right)}-d_{0}\right)\varepsilon \cdot \frac{1}{2\left(a-bp\right)}d\varepsilon \\ +\gamma q\int_{0}^{q}\frac{1}{2\left(a-bp\right)}d\varepsilon +pq\int_{q}^{2\left(a-bp\right)}\frac{1}{2\left(a-bp\right)}d\varepsilon \\ =\left(\text{-}w_{0}+p\right)q+\frac{3\left(p-\gamma \right)q^{2}}{8\left(a-bp\right)}\left(\frac{p-vp_{m}}{p_{m}\left(u-v\right)}-d_{0}\right)-\frac{\left(p-\gamma \right)q^{2}}{2\left(a-bp\right)} \end{array}$$ (22)

In this supply mode, we need to use only the retailer’s optimal purchase volume and optimal price to derive the correlation function from the above formula and set its result equal to 0.

At this point, the retailer’s optimal pricing formula *p* is then

$$\begin{array}{l} \frac{\partial \Pi_{RF}}{\partial p}=q+\frac{3q^{2}}{8\left(a-bp\right)}\left(\frac{2p-vp_{m}-\gamma }{p_{m}\left(u-v\right)}-d_{0}\right)+\frac{3\left(p-\gamma \right)bq^{2}}{8{\left(a-bp\right)}^{2}}\left(\frac{p-vp_{m}}{p_{m}\left(u-v\right)}-d_{0}\right)\\ -\frac{q^{2}}{2\left(a-bp\right)}-\frac{\left(p-\gamma \right)bq^{2}}{2{\left(a-bp\right)}^{2}}=0 \end{array}$$ (23)

The solution for *p* is the retailer’s optimal price.

Then,

$$\frac{\partial \Pi_{RF}}{\partial q}=\text{-}w_{0}+p+\frac{3\left(p-\gamma \right)q}{4\left(a-bp\right)}\left[\left.\frac{p-vp_{m}}{p_{m}(u-v)}-d_{0}\right]\right.-\frac{\left(p-\gamma \right)q}{\left(a-bp\right)}=0$$ (24)

The solution for *q* is the retailer’s optimal purchase amount.

## 5 Numerical example

Under nonessential demand patterns, the relationships among the variables involved in the retailer’s design and planning of supply strategies to determine the profit-maximizing model are very complicated. There is no specific solution for the optimal purchase quantity or the optimal price. We verify the optimal solution of the model through numerical examples using these models to test whether they truly reflect real-world PST problems for different demand patterns.

We assume that the production cost of a product produced by company J is 70 and that the highest retail price of the product in the market is 100. Here, the retail price of the product sold by the retailer to the individual customer needs to satisfy the following inequality: 70 < *p*≤100. The basic parameters are set as follows: *a* = 90, *w*_0_ = 70, *d*_0_ = 0.2, *b* = 0.8, *p*_*m*_ = 100, *u* = 0.9, and *v* = 0.8.

### 5.1 Off-invoice mode

We replace the functions in Eqs (15) and (16) with the basic parameter settings listed above and solve them with Mathematica 8.0 to obtain the optimal purchase quantity and the optimal price in the off-invoice mode. We compute *α* in increments of 0.5 in the interval (0.5, 3) to understand how the manufacturer’s rediscount to retailer *α* affects the retailer’s optimal order quantity and price. The validity of the model is verified by the fact that the result of the calculation satisfies the constraint 70 < *p*≤100 (see Table 2).

**Table 2:** Optimal order quantities and optimal prices under the off-invoice mode.

*a*	*p*	*q*
0.5	80.65	12.74
1.0	80.66	12.73
1.5	80.67	12.73
2.0	80.67	12.72
2.5	80.68	12.72
3.0	80.69	12.71

**Fig 2:**
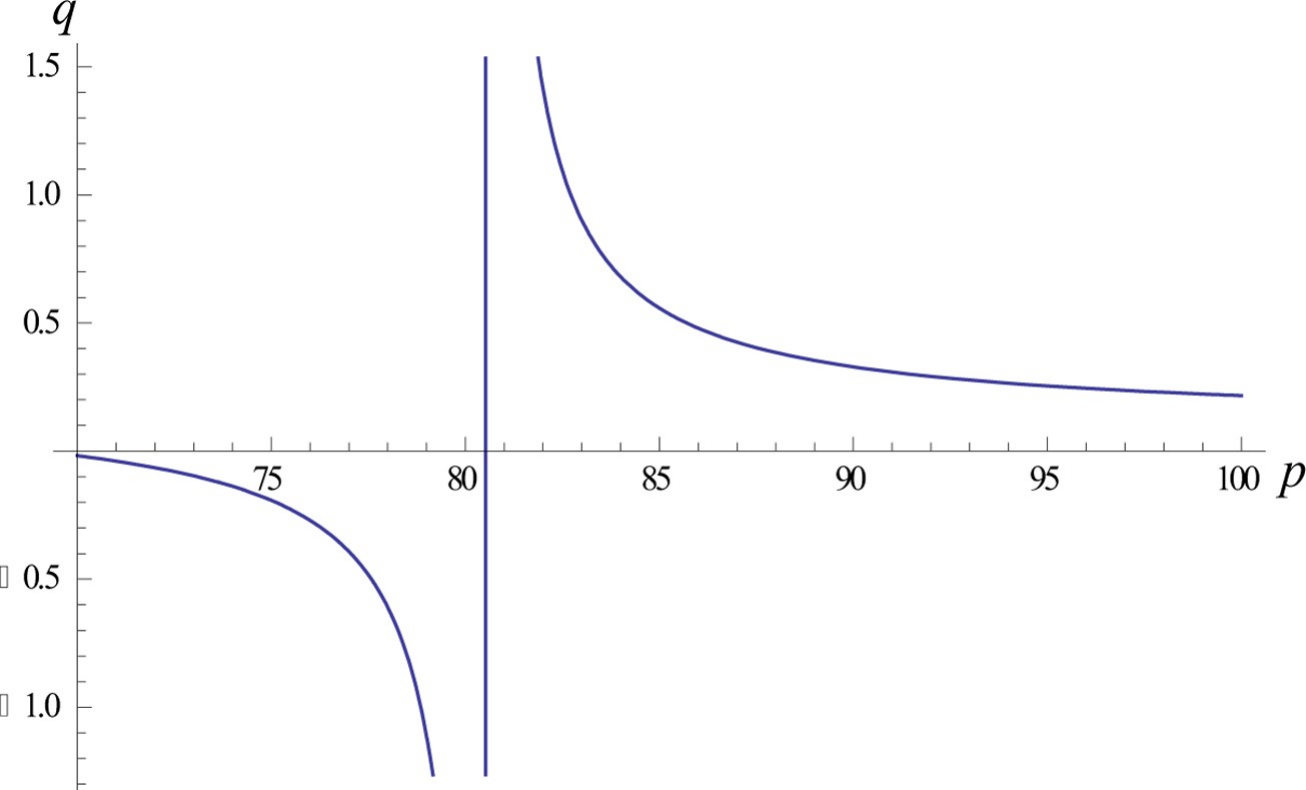
Trend graph of optimal pricing with the optimal order quantity in the off-invoice mode.

We analysed the optimal profit in the off-invoice mode, as shown in Fig 2. In the off-invoice mode, the relationship between the optimal price and the optimal order quantity is hyperbolic. The apex of the upwards-trending curve represents the most advantageous pricing strategy for the retailer to achieve optimal profit. However, if the product is priced within the interval (70,78), the curve is trending downwards, and the value of the order quantity *q* is negative, which is not in line with the law of merchandising; from this, it can be inferred that at this point, the retailer is selling at a loss.

### 5.2 Scan-back mode

We replace the functions in Eqs (19) and (20) with the basic parameter settings described and solve them with Mathematica 8.0 to obtain the optimal purchase quantity and the optimal price in the scan-back mode. We compute *β* in increments of 0.5 in the interval (0.5, 3) to understand how the manufacturer’s rerebates to retailer *β* affects the retailer’s optimal order quantity and price. The validity of the model is verified by the fact that the result of the calculation satisfies the constraint 70 < *p*≤100 (see Table 3).

**Table 3:** Optimal order quantities and optimal prices under the scan-back mode.

*β*	*p*	*q*
0.5	96.42	0.05
1.0	96.22	0.05
1.5	96.02	0.05
2.0	95.82	0.05
2.5	95.61	0.05
3.0	95.42	0.06

**Fig 3:**
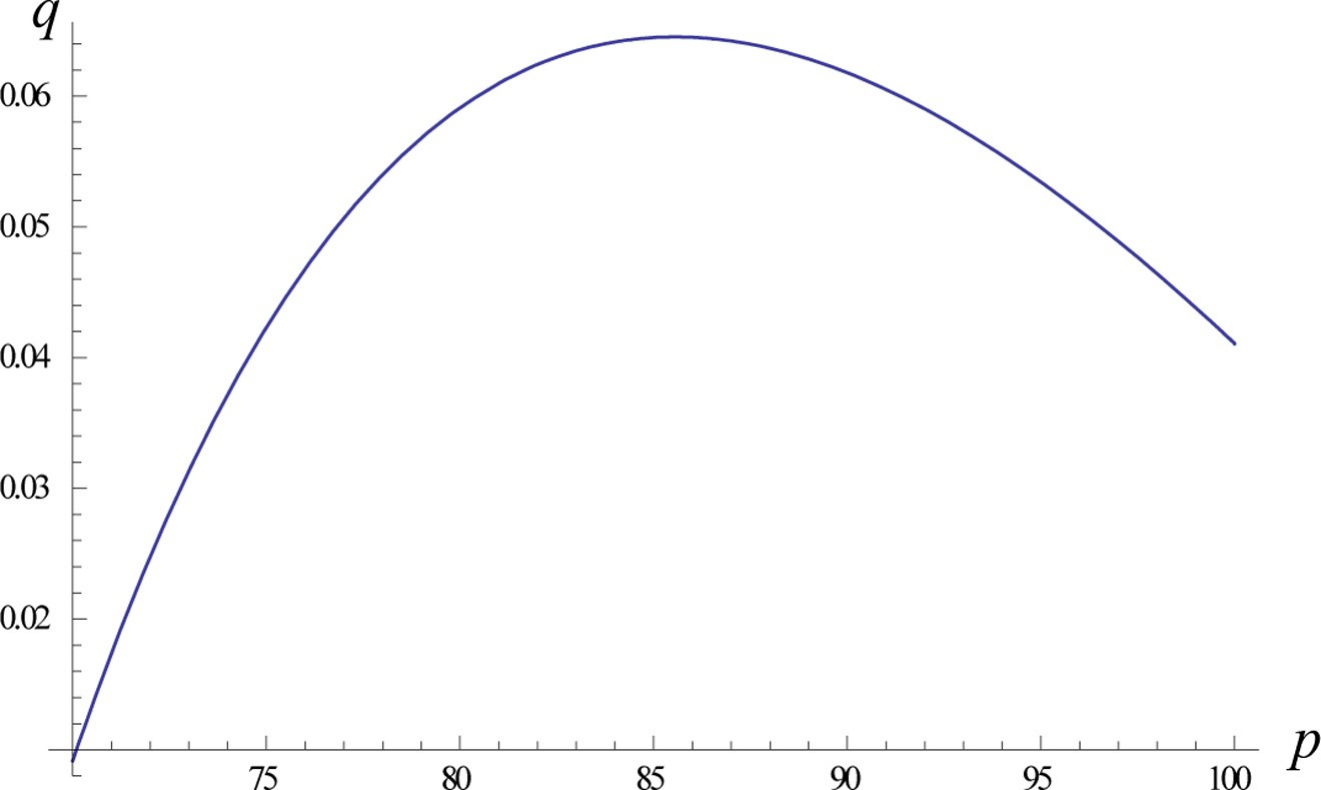
Trend graph of optimal pricing with the optimal order quantity in the scan-back mode.

We analysed the optimal profit in the scan-back mode, as shown in Fig 3. In the scan-back mode, the relationship between the optimal price and the optimal order quantity is a backwards parabola. The apex of the upwards-trending curve represents the most advantageous pricing strategy for the retailer to achieve optimal profit.

### 5.3 Return credit mode

We replace the functions in Eqs (23) and (24) with the basic parameter settings described above and solve them with Mathematica 8.0 to obtain the optimal purchase quantity and the optimal price in the scan-back mode.

We compute γ in increments of 1 in the interval (64, 69) to understand how the manufacturer’s rerecyclion to the retailer γ affects the retailer’s optimal order quantity and price. The validity of the model is verified by the fact that the result of the calculation satisfies the constraint 70 < *p*≤100 (see Table 4).

**Table 4:** Optimal order quantities and optimal prices under the return credit mode.

γ	*p*	*q*
64	70.05	0.24
65	70.04	0.24
66	70.03	0.23
67	70.02	0.23
68	70.02	0.23
69	70.01	0.23

**Fig 4:**
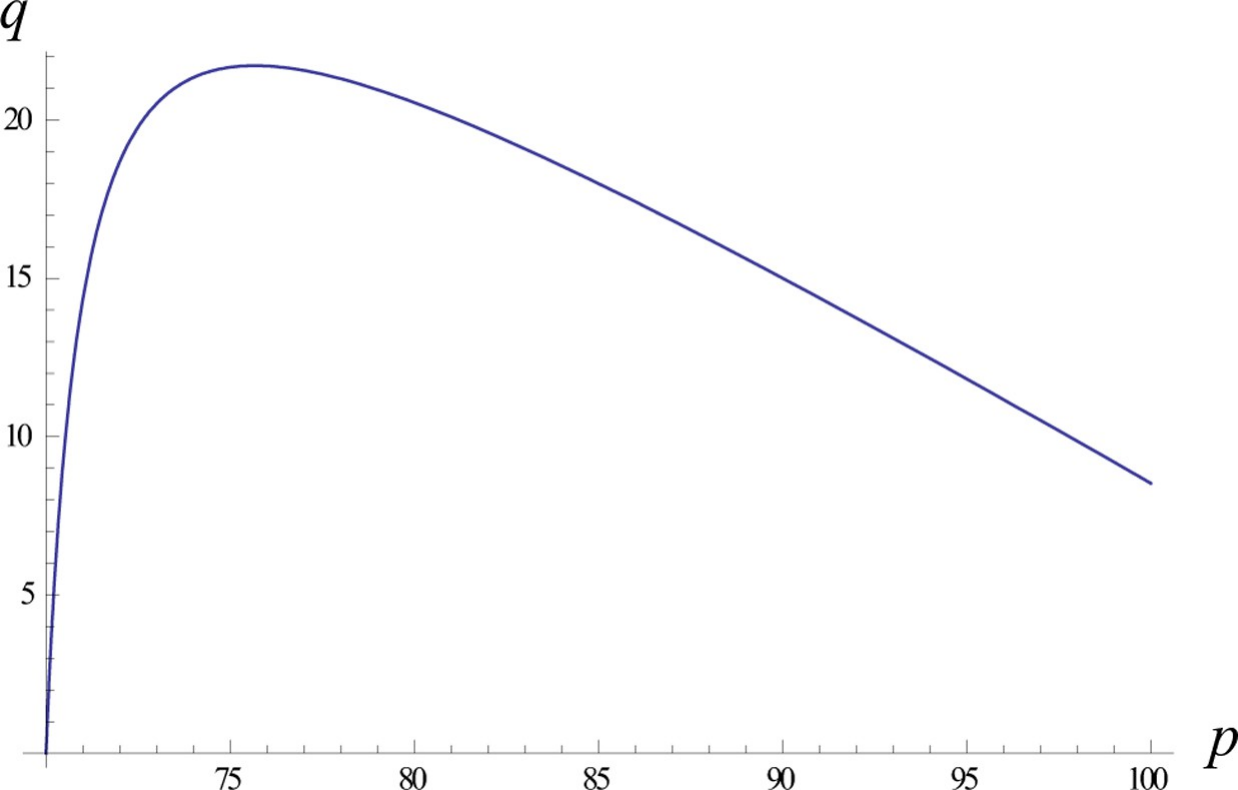
Trend graph of optimal pricing with the optimal order quantity in the return credit mode.

We analysed the optimal profit in the return credit mode, as shown in Fig 4. In the return credit mode, the relationship between the optimal price and the optimal order quantity is a backwards parabola. The apex of the upwards-trending curve represents the most advantageous pricing strategy for the retailer to achieve optimal profit.

## 6 Conclusions

Since the start of the 21st century, advances in industrial technology, information technology, and management systems have led to significant growth in social productivity. Additionally, the development of the Internet+, Internet of Things+, cloud computing, artificial intelligence and other innovative technologies has driven a fresh wave of scientific and technological revolutions and industrial transformations. The advancement of science and technology has engendered a transformation not only in people’s production and lifestyle but also in their requirements—from resolving basic issues of sustenance to fulfilling contemporary spiritual aspirations. Therefore, exploring customer demand during this period is a great challenge for companies. Enterprises must continually innovate with technology, create new products and enhance their services to meet consumer demand. A balance should also be found between oversupply and undersupply to maximize the profitability of products. Given such opportunities and challenges, it is important for manufacturers and retailers to design and plan product supply strategies for individual and group customers according to different patterns of customer demand. This paper addresses this research problem by applying economic theory and optimization theory to establish numerical ranges and function graphs under different demand patterns, which is also the theoretical contribution of this paper.

In this paper, we studied the retailer-to-individual customer optimal supply strategy problem under a nonessential demand pattern. We analysed the optimal supply strategies in three modes—the off-invoice mode, scan-back mode, and unsold recycling mode—and constructed corresponding mathematical models. By comparing the three models, we find that the retailer’s profits will be maximized under the unsold recycling mode.
